# Supplementary material for: Oclacitinib and Myxoma Virus Therapy in Dogs with High-Grade Soft Tissue Sarcoma
Source: Biomedicines. 2023 Aug 23;11(9):2346. doi: 10.3390/biomedicines11092346 (PMC10525839; doi:10.3390/biomedicines11092346)
Supplement: Supplementary file 1 [file biomedicines-11-02346-s001.zip › Table S1.pdf]

Table S1. Clinical data lists. A list of data collected to screen dogs for any adverse events associated with O+MYXVΔSERP2 treatment is provided. The Veterinary Comparative Oncology Group criteria were used to analyze the data collected from each patient at multiple time points during the study. No adverse events > Grade 1 were observed in patients treated pre-operatively with oclacitinib and post-operatively with MYXVΔSERP2.

Physical examination assessments	Complete blood count	Serum biochemistry profile	Urinalysis
attitude	packed cell volume	glucose	refractometer specific gravity
awareness	hemoglobin concentration	blood urea nitrogen	pH
mobility	hematocrit	creatinine	nitrite
hydration	red blood cell concentration	phosphorus	protein
body condition score	mean corpuscular volume	calcium	glucose
body weight	red cell distribution width	magnesium	ketones
temperature	mean corpuscular hemoglobin concentration	total protein	bilirubin
heart rate	cellular hemoglobin concentration mean	albumin	blood
respiratory rate	platelet concentration	globulin	color
pulse	mean platelet volume	albumin to globulin ratio	clarity
auscultation of heart, lungs & intestinal tract	total nucleated cell concentration	cholesterol	cytologic examination of the sediment
hair coat	neutrophil concentration	creatine kinase	
oral cavity	lymphocyte concentration	total bilirubin	
eyes	monocyte concentration	alkaline phosphatase	
ears	eosinophil concentration	alanine aminotransferase	
palpation of internal organs, anal glands & lymph nodes	basophil concentration	aspartate aminotransferase	
tumor measurements	large unidentified cell concentration	gamma-glutamyl transferase	
	cytologic evaluation of a blood smear	sodium	
		potassium	
		chloride	
		bicarbonate	
		anion gap	
