# Supplementary figures and images for: Oclacitinib and Myxoma Virus Therapy in Dogs with High-Grade Soft Tissue Sarcoma
Source: Biomedicines. 2023 Aug 23;11(9):2346. doi: 10.3390/biomedicines11092346 (PMC10525839; doi:10.3390/biomedicines11092346)

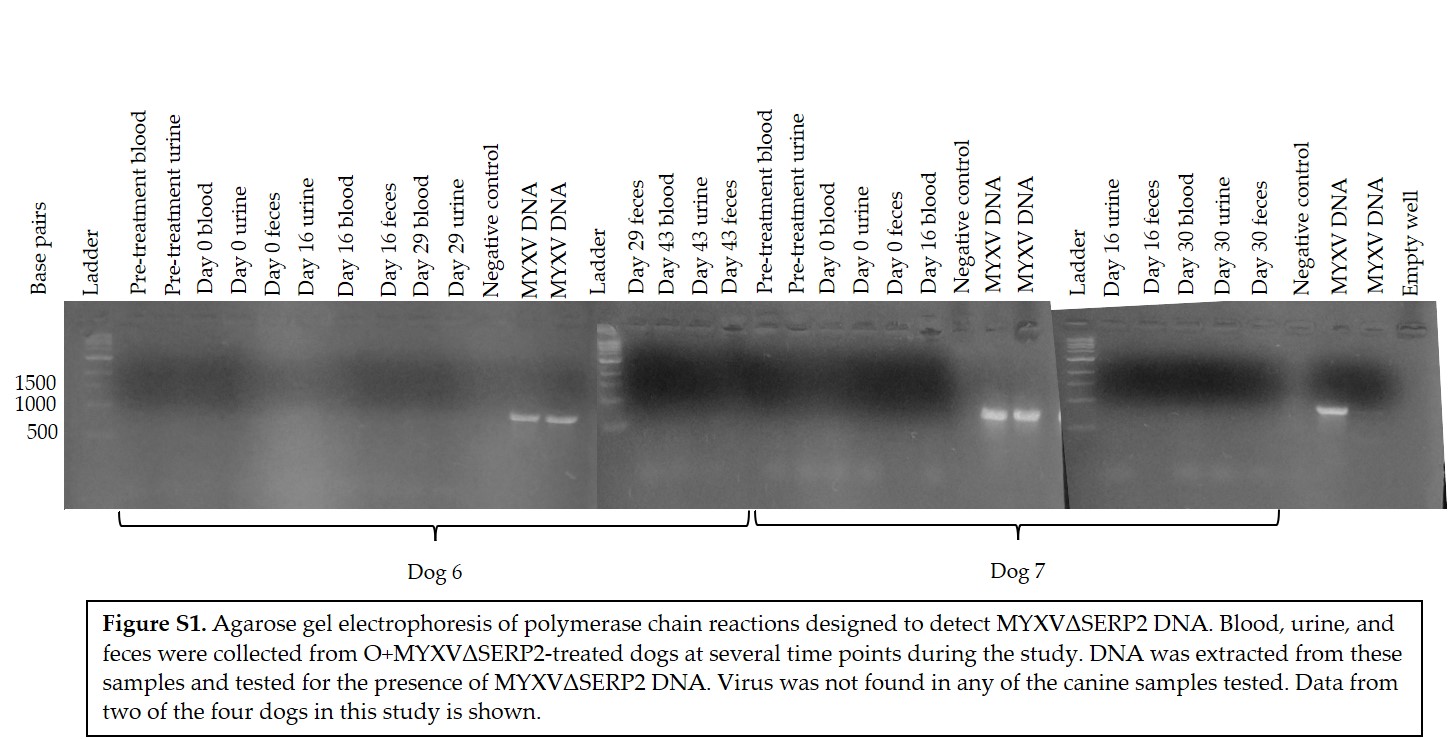

Supplement: Supplementary file 1 [file biomedicines-11-02346-s001.zip › Figure S1.jpg]

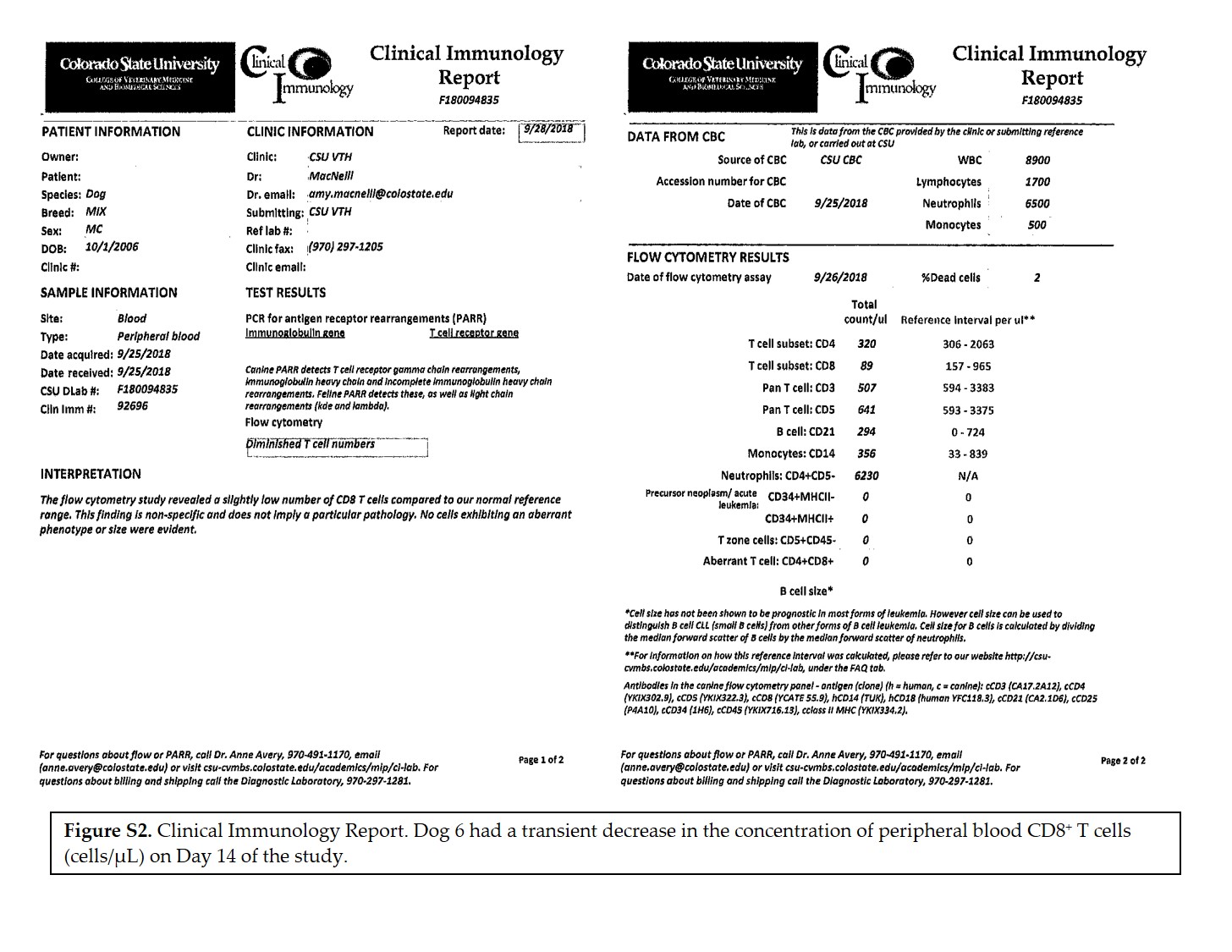

Supplement: Supplementary file 1 [file biomedicines-11-02346-s001.zip › Figure S2.jpg]
